# Case Report: Complex Congenital Brain Anomaly in a BBxHF Calf–Clinical Signs, Magnetic Resonance Imaging, and Pathological Findings

**DOI:** 10.3389/fvets.2021.700527

**Published:** 2021-09-22

**Authors:** Neeltje J. Veenema, Koen M. Santifort, Nienke W. Kuijpers, Anne Seijger, Peter R. Hut

**Affiliations:** ^1^Department of Population Health Sciences, Faculty of Veterinary Medicine, University of Utrecht, Utrecht, Netherlands; ^2^Evidensia Animal Hospital Arnhem, Arnhem, Netherlands; ^3^Department of Diagnostic Imaging, Faculty of Veterinary Medicine, University of Utrecht, Utrecht, Netherlands

**Keywords:** bovid, cerebellar ataxia, corpus callosum agenesis, hydrocephalus, porencephaly

## Abstract

This case report describes the clinical signs, magnetic resonance imaging (MRI) findings and associated (histo)pathological findings in a crossbred Belgian Blue calf with congenital complex brain anomaly. The calf was presented with non-progressive signs (including cerebellar ataxia) since it was born, suggestive of a multifocal intracranial lesion. A congenital anomaly was suspected and after hematology, biochemistry, serology, and cerebrospinal fluid analysis, a magnetic resonance imaging study was performed. The following suspected abnormalities were the principal changes identified: severe hydrocephalus, porencephaly, suspected partial corpus callosum agenesis (CCA), and increased fluid signal between the folia of the cerebellum. Post-mortem examination predominately reflected the MRI findings. The origin for these malformations could not be identified and there was no evidence of a causative infectious agent. Corpus callosum abnormalities have been reported in bovids before and have been linked to bovine viral diarrhea virus (BVDV) infections, as have several other central nervous system anomalies in this species. In this case, BVDV was deemed an unlikely causative agent based on serology test results and lack of typical histopathological signs. The etiology of the congenital anomaly present in this bovine calf remains unknown.

## Background

Central nervous system (CNS) anomalies in cattle are commonly reported in cases of *in utero* viral infections such as bovine viral diarrhea virus (BVDV) infections (1–3). Several bovine congenital anomalies related to viral infections, such as exencephaly, hydrocephalus, meningoencephaloceles, and diplomyelia have been characterized post-mortem (2, 4–6). Magnetic resonance imaging (MRI) is the preferred diagnostic imaging modality to visualize and evaluate structures of the CNS and has been used to detect CNS anomalies in bovids (6, 7). The normal MRI neuroanatomy of calves has been described in Schmid et al. (8). This case report describes the clinical signs, magnetic resonance imaging findings and pathological findings in a bovine calf with a congenital complex brain anomaly.

## Case Presentation

### History

A 4-week-old male crossbred Belgian Blue calf was presented to the Farm Animal Health clinic of the Faculty of Veterinary Medicine, Utrecht, The Netherlands for investigation of non-progressive problems with ambulation since birth. No abnormalities or specific peculiarities were evident on review of the history pertaining to the gestational, peri- or post-natal period and parturition itself was uneventful. The status of the calf's original farm for Bovine Viral Diarrhea virus (BVDV) was BVDV negative; all cattle are vaccinated with an inactivated vaccine (Bovilis BVD, MSD, the Netherlands). The farm also tested negative for Bovine Herpes virus and Leptospirosis. For Johne's disease the farm tested status B. *Salmonella* was not suspected (Royal GD, Deventer, The Netherlands).

### Clinical Examination

General physical examination findings were unremarkable. Neurological examination (9) resulted in the following findings: the calf appeared bright and responsive and was able to stand on its own. However, when attempting to stand up, the calf took a broad-based stance while noticeably swaying the head and trunk. The head was positioned low, close to the ground most of the time. The head and truncal sway became markedly worse upon ambulation. Ataxia of all limbs was evident with mild hypermetric movements, mainly the thoracic limbs (see Supplementary Video 1). There were no signs of paresis. The calf used the walls of the pen for support but frequently fell over when support was not available. This was especially noticeable when the calf was prompted to circle. Forced deviation of the head to either side resulted in increased swaying. Postural responses, tail pull and crossed limb-tests frequently resulted in stumbling. The menace response was present bilaterally and the calf stepped over obstacles in the obstacle test. When testing the pupillary light reflexes, pupillary constriction was present but slow (3 s to full constriction). Indirect pupillary light reflexes were present. Other cranial nerve function tests did not reveal abnormalities, nor did examination of the spinal reflexes. The remainder of the neurological examination was unremarkable. The neuroanatomical localization was multifocal intracranial involving the cerebellum and rostral brainstem (diencephalon/mesencephalon).

Clinical examination was repeated approximately once a month until the calf was euthanized at 10 months of age. No abnormalities in food or water intake were noticed during that time. The outcome of the clinical examinations remained similar (i.e., clinical signs were non-progressive or static).

## Diagnostics

Hematology and biochemistry tests revealed no significant abnormalities. Specifically, sodium levels were found to be within reference range (135 mmol/L; 135–150 mmol/L). At 40 days of age, serological tests were performed at the Animal Health Service (Royal GD, Deventer, The Netherlands) for BVDV antigen and antibodies, Schmallenberg virus antibodies, Bluetongue virus antibodies and *Neospora* antibodies. Only BVDV antibodies were detected. At 12 weeks of age, serology was repeated on serum for BVDV antibodies and antigen; both were not detected.

Cerebrospinal fluid was collected at 85 days of age and analyzed at the University Veterinary Diagnostic Laboratory (UVDL, Utrecht, The Netherlands). Protein concentration was within range at 0.22 g/L (reference limit <0.4–0.67 g/L) (10, 11). The total nucleated cell count (TNCC) was elevated at 33 cells /μL (reference limit <10 cells/μL) (10). Cytology revealed a mixed pleocytosis. Bacterial culture performed at the Veterinary Microbiology Diagnostic Center (VMDC, Utrecht, The Netherlands) was negative.

Differential diagnoses of consideration principally included complex congenital brain malformations or inflammatory lesions (based on CSF findings). The suspicion of brain malformations was further corroborated by additional diagnostic tests and follow-up. To evaluate the brain *in vivo*, an MRI examination was deemed the next diagnostic step.

## Outcome and Follow-Up

### Magnetic Resonance Imaging

At 55 days of age a magnetic resonance imaging (MRI) study of the head was performed (1.5 Tesla Philips Ingenia, Philips Healthcare, Eindhoven, The Netherlands) under general anesthesia. Premedication consisted of xylazine 8 mg intramuscular and detomidine 1 mg intravenously, followed by anesthesia induction with propofol 160 mg intravenously to facilitate tracheal intubation. Anesthesia was maintained with sevoflurane 3% gas-inhalation. A body coil was applied at the top of the head. The MRI included the following sequences: transverse T2-weighted turbo spin-echo (TR 4433, TE 100, slice thickness 6 mm, interslice gap 6 mm), sagittal T2-weighted turbo spin echo (TRE 5032, TE 100, slice thickness 5 mm, interslice gap 5 mm) transverse T1-weighted turbo spin-echo before and after intravenous contrast administration; gadoterate meglumine 0.2 ml/kg (Dotarem, etc) (TR 580, TE 15, slice thickness 6 mm, interslice gap 6 mm) transverse fluid-attenuated inversion recovery (FLAIR; TR 11000, TE 140, TI 2800, slice thickness 6 mm, interslice gap 6 mm etc.), transverse T2^*^fast-field echo (TR 618, TE 14, slice thickness and interslice gap 6 mm), transverse DWI (TR 4094, TE 90, slice thickness and interslice gap 4 mm, B1000) and ADC map (TR 4094, TE 90, slice thickness and interslice gap 4 mm) sequences. Field-of view was adapted per sequence to optimize image quality.

**Figure 1:**
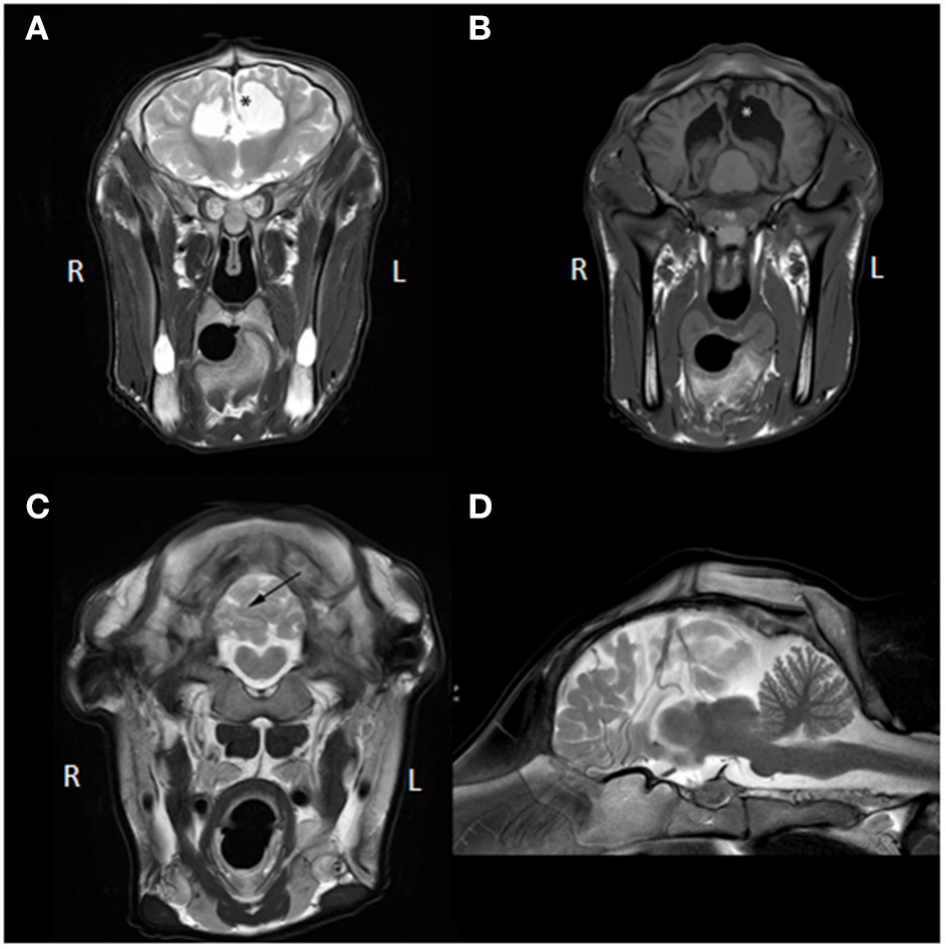
**(A)** Transverse T2W MR images at the level of the third ventricle, **(B)** transverse T1W MR images at the level of the caudal aspect of the lateral ventricles and rostral colliculi of the mesencephalon, **(C)** transverse T2W MRI image at the caudal aspect of the cerebellum, **(D)** mid-sagittal T2W MR image. The asterisk (*) in **(A,B)** demarcates the communication between the left lateral ventricle and the subarachnoid space and represents the porencephaly. Also, in **(A)**, communication between the left lateral and right lateral ventricle is visible due to partial absence of the septum pellucidum and no corpus callosum is identifiable. In **(B)**, fusion of the rostral colliculi is appreciated. In **(C)**, the black arrow points toward the irregular and apparently incomplete separation between right and left cerebellar hemisphere (not confirmed on post-mortem examination; likely represents *in situ* displacement). Partial herniation of the left hemisphere over the midline into the right hemisphere is visible (not confirmed on post-mortem examination; likely represents *in situ* displacement). In **(D)**, the corpus callosum and mesencephalic aqueduct are hard to identify and increased fluid signal between cerebral gyri and cerebellar folia is seen, which can also be appreciated in **(A–C)**.

**Figure 2:**
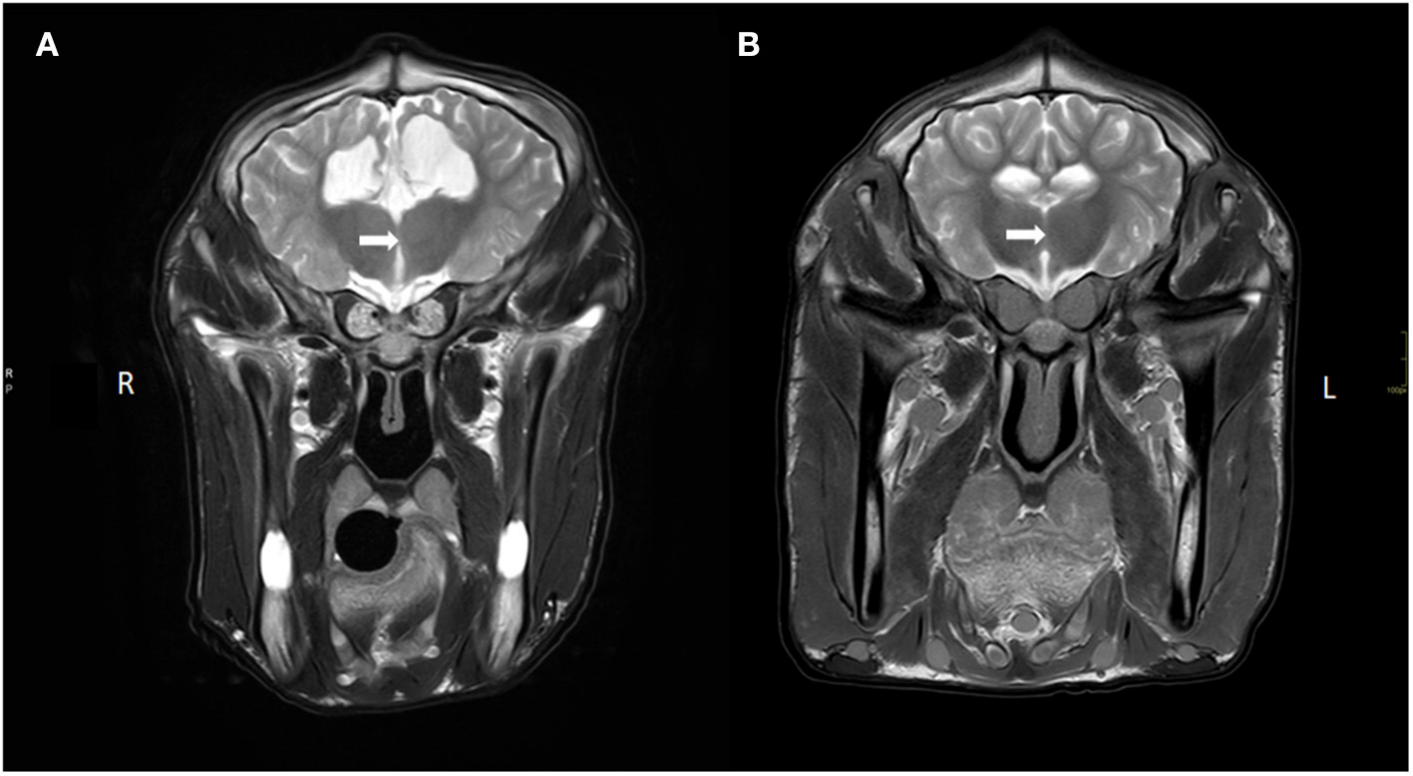
Transverse T2W images of the brain at the level of interthalamic adhesion of **(A)** our current case with a hypoplastic/absent interthalamic adhesion (white arrow) in comparison with the interthalamic adhesion in **(B)** of a calf without brain lesions (at 7 months of age). Also note the ventriculomegaly and thin white matter tracts (corona radiata), thin cortex and increased CSF signal ventral to the thalamus in **(A)** compared to **(B)**.

Several abnormalities were identified (see Figure 1): the ventricular system was severely enlarged with asymmetric dilation of the lateral ventricles bilaterally and dilation of the third ventricle with abnormal shape and partial absence of the septum pellucidum. The corpus callosum was only partially visualized. The interthalamic adhesion was subjectively small, which can be appreciated when comparing the images to those of another calf taken at 7 months of age (see Figure 2). Parts of the left dorsomedial cerebral hemisphere and left lateral ventricle ependymal lining were absent. This resulted in a connection between the subarachnoid space and the ventricles and an increase in subarachnoid space width at the dorsal midline; this was less extensive toward the lateral aspects of the cerebrum. In addition, supracollicular fluid accumulation was noticeable and the rostral colliculi were at least partially fused in the midline. The mesencephalic aqueduct was difficult to visualize in its entirety and obstruction could not be ruled out. An increased fluid signal between cerebellar folia, and an undefined separation of the cerebellar hemispheres were also noted and crossing of the left hemisphere to the right was suspected due to partial absence of the vermis. Signal characteristics and definition of white and gray matter, sulci and gyri, and other gross structures were not deemed abnormal, and no abnormal contrast enhancement was noted. The ventricle: cerebrum ratio (VC ratio) was calculated from transverse images according to the method described by Tsuka et al. The VC ratio was 0.18 (18%) which was consistent with hydrocephalus [VC ratio ≥0.15; the reference for Holstein-Friesian cattle is reported to be 0.082 ± 0.030 (0.037–0.151) on the transverse plane] (12).

These findings were compatible with a congenital complex brain anomaly including internal hydrocephalus (possibly obstructive at the level of the mesencephalic aqueduct) with left cerebral porencephaly, (partial) corpus callosum agenesis or hypoplasia, cerebral hypoplasia, cerebellar vermis hypoplasia, and malformation.

### Post-mortem Examination

**Figure 3:**
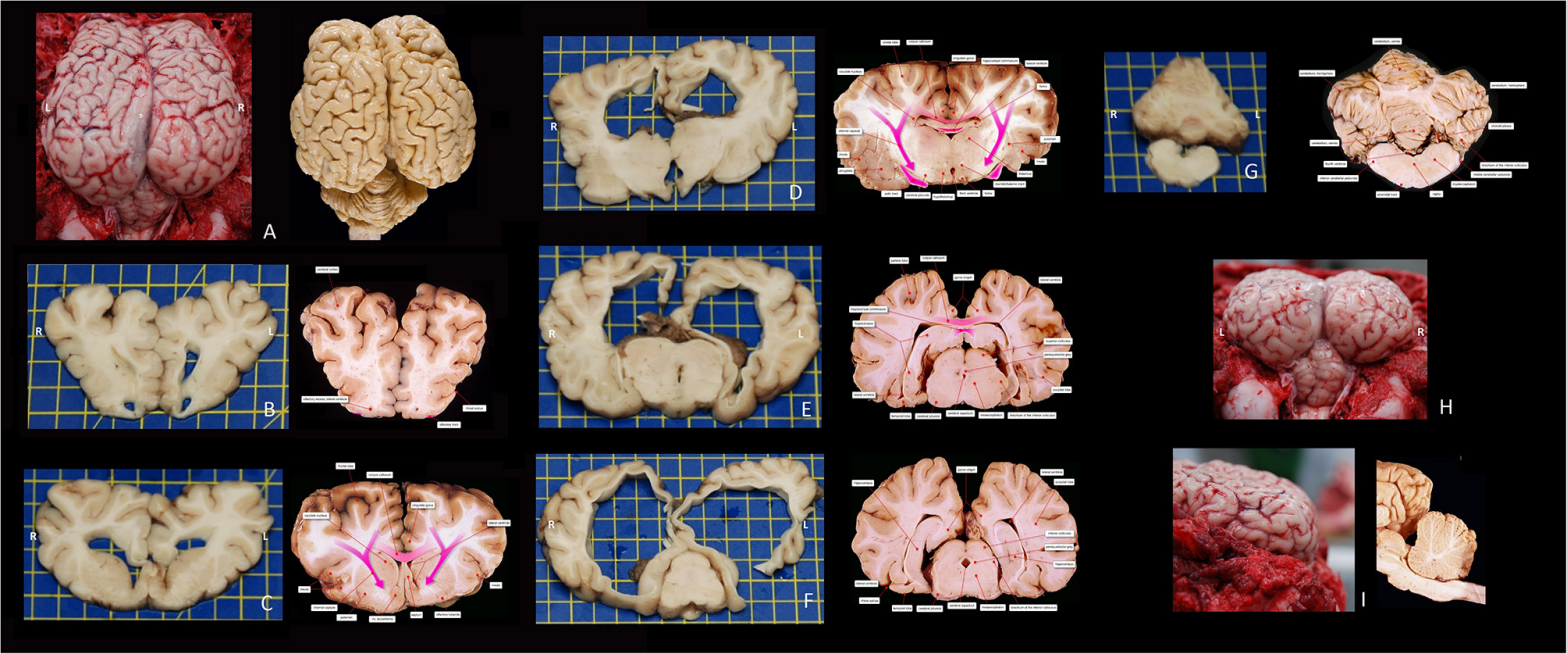
Macroscopic photographs of the bovine encephalon of this case report and corresponding reference images (fixated in formalin) at approximately the same level–courtesy of dr. FG Wouterlood and dr. P. Voorn, Dept Anatomy and Neurosciences, AmsterdamUMC, VU, Amsterdam, The Netherlands. **(A)** Dorsal view of the cerebrum and cerebellum *in situ* after removal of the dorsal and caudal parts of the skull and dura mater. **(B)** Rostrocaudal view of a transverse slab at the level of the olfactory recesses. **(C)** Rostrocaudal view of a transverse slab at the level of the septal area. **(D)** Rostrocaudal view of a transverse slab at the level of the interthalamic adhesion. **(E)** Rostrocaudal view of a transverse slab at the level of the rostral mesencephalon. **(F)** Rostrocaudal view of a transverse slab at the level of the caudal mesencephalon. **(G)** Rostrocaudal view of a transverse slab at the level of the cerebellum, fourth ventricle, and medulla. **(H)** Caudorostral view of the cerebrum and cerebellum *in situ* after removal of the dorsal and caudal parts of the skull and dura mater. **(I)** laterolateral view of the cerebrum *in situ* after removal of the dorsal and caudal parts of the skull and dura mater. The anatomical terms differ from those of the Nomina Anatomica Veterinaria, as the images are composed and labeled by human neuroanatomists/-scientists.

The quality of life of the calf was considered good (based on general examination and general function of the animal) despite the poor prognosis for the neurological disorder. The clinical presentation of non-progressive signs of a multifocal intracranial lesion remained unchanged over a 10-month period. At 10 months of age the calf was euthanized by intravenous administration of 100 mL pentobarbital (Euthanimal 40%, Alfasan, the Netherlands) and a post-mortem examination was performed at the Veterinary Pathology Diagnostic Center (VPDC, Utrecht, The Netherlands). This revealed revealed diffuse, moderately enlarged hemispheres most prominently on the left side, covered by diffuse moderately edematous leptomeninges (see Figure 3). Dilation of both lateral ventricles, hydrocephalus. was evident. Defects were noted in the left lateral ventricular wall, in the white matter and in the cortex in the parieto-occipital region. This resulted in a connection between the subarachnoid space and the ventricles (porencephaly; compare Figure 1B with Figure 3E). Temporal and occipital lobe cortex and white matter were severely reduced in thickness to <5 mm. The olfactory lobe recess and third and fourth ventricular lumens were bilaterally moderately dilated. Rostrally and around the middle part of the lateral ventricles there was a thin tract of white matter crossing the midline. This was interpreted to represent hypoplastic rostral, genu, and part of the body of the corpus callosum. The rest of the corpus callosum, i.e., the caudal part of the body and splenium could not be identified. The caudate nuclei were compressed, as were the fornix, lamina terminalis and rostral commissure. The hippocampus was present but dysplastic and hypoplastic. The diencephalon was reduced in size and dysplastic. The mesencephalic aqueduct could not be identified at the level of the fused rostral colliculi. Otherwise, the mesencephalon, pons, and medulla oblongata did not show visible abnormalities macroscopically. The cerebellum showed a mild reduction in size especially in the cerebellar hemispheres without coning through the foramen magnum. The suspected left to right hemisphere herniation noted on the MRI examination were not evident; it was suspected that displacement of the cerebellar tissue *in situ* resulted in these MRI findings and that post-mortem removal of skull structures and release of CSF resulted in a more normal position and an anatomically fairly normal cerebellum. Microscopy revealed a severely thinned cerebral cortex (suspected pressure atrophy) with diffuse spongy change in gray matter and subcortical white matter (edema). The white matter also showed mild astrogliosis and astrocytosis. The neuropil of the brainstem showed a multifocal spongy change and mild astro- and microgliosis. Degenerative neurons were seldomly seen throughout the histological samples of the brainstem. The cerebellar folia were intact. Moreover, no microscopic evidence of cerebellar hypoplasia was found; no signs of disorganization of the granular layer molecular layer or white matter and no obvious loss or degenerative changes in Purkinje neurons was noticed. In addition, there was no evidence for a causative infectious agent and there was a lack of typical signs suggestive for viral encephalitis (e.g., perivascular cuffing or neuronal inclusion bodies). In conclusion, the main findings on post-mortem examination were partial agenesis and hypoplasia of the corpus callosum, fornix and septum pellucidum, obstructive internal hydrocephalus and porencephaly, which morphologically reflected the MR images (see Figures 1, 3). The origin for these congenital anomalies could not be identified.

## Discussion

Congenital bovine CNS malformations have been described in the literature to a certain extent and congenital malformations are divided into the following categories: defects of neural tube closure; defects of forebrain induction; neuronal migration disorders and sulcation defects; disorders of proliferation or size; encephaloclastic defects; cerebellar- (or caudal cranial fossa-) and spinal malformations; congenital hydrocephalus and cysts (13). Corpus callosum agenesis (CCA), partial or complete, would be categorized as a defect of forebrain induction. The porencephaly can be categorized as an encephaloclastic defect (13).

Although rare (partial) corpus callosum agenesis has been previously described in cattle (1, 14, 15). Buck et al. described a total absence of the corpus callosum, septum pellucidum and hippocampal commissure in 2 calves. The fornices were present but separated and necropsy further demonstrated an internal hydrocephalus, micrencephaly, and a CSF-filled cyst in the longitudinal cerebral fissure dorsal to the thalamus (14). Kisipan et al. described a calf with a deformed face and holoprosencephaly, a hypoplastic corpus callosum and underdeveloped hippocampal formation (15). Interestingly on clinical examination no specific neurological signs were noted (14, 15). In those case reports, pathogenesis and etiology remain unknown and no evidence of inflammation or genetic disorders was found as a cause for the brain anomaly (14, 15). In contrast to the few reports of bovine CNS anomalies, human congenital CNS anomalies have been widely reported. However, these reports also frequently can not provide a definite conclusion about the etiology of the malformations (16–19). Several of the reported anomalies found in humans overlap with the anomalies described in our bovine case (16–19), for example the dysplastic hippocampi that were described to be present in all types of cortical development malformations in human literature (20). The prognostic relevance of partial CCA in association with the absence of commissures of the hippocampus or fornix is thought to be representative of a more severe form of a cerebral anomaly than CCA by itself (20). CCA can interfere with the regular process of hippocampal formation and growth, resulting in its underdevelopment; in humans this can account for certain learning and memory difficulties (21). No specific pattern of hippocampal malformation however has been associated with a particular type of brain anomaly (20–22). In humans the neurologic examination is more comprehensive and involves the performance of directed tasks and evaluation of speech language capabilities, but this cannot be performed in other species rendering an accurate clinical diagnosis more difficult. Sentence comprehension skills have been shown to be adversely affected in humans with CCA (23). This obviously cannot be assessed in animals, but other behavioral signs may be indicative for these types of cerebral malformations. In this case, no behavioral abnormalities were noticed and the calf's behavior and consciousness were both deemed normal despite MRI and post-mortem confirmation of forebrain involvement. This may be best explained by the limitations of the bovine clinical examination.

Congenital anomalies can have several causes such as exposure to toxins, nutritional deficiencies, infections, genetic mutations and degenerative diseases. In some cases the etiology remains unexplained (1, 2). In the context of infectious causes, *in utero* infection with viral agents is linked to bovine brain anomalies such as porencephaly, hydranencephaly, hydrocephalus, cerebellar hypoplasia, caused by viruses including Bovine Viral Diarrhea virus, Schmallenberg virus, Akabane virus, Cache Valley virus and Aino virus (*Orthobunya virus*), Bluetongue virus and Chuzan virus (*Orbivirus*), and Wesselbron virus (*Flavivirus*) (1, 2, 13). But no brain anomalies are pathognomonic for specific viruses and diagnosing a viral cause based on gross lesions is ill-advised. The complex anomaly described in this case has several features that have not been described in combination as being cause by any of these viruses (1, 13). The macroscopic pathological and MRI findings of a complex brain anomaly could theoretically have been caused by a transplacental BVDV infection between day 90 and 150 of gestation (1) and serological tests were performed even though a BVDV infection was deemed unlikely based on the status of the farm. Serological tests for both BVDV antigen and antibodies were performed, and antibodies for BVDV were found at 40 days of age. The presence of antibodies can be explained through either colostrum-derived antibodies (transient infection or vaccination of the dam), a transient infection of the calf, or an intra-uterine infection between 75 and 170 days of gestation (7). The farm this calf originated from employed vaccination prevention strategies for BVDV for over 25 years and bovids that have been vaccinated can develop titers which can lead to the detection of these maternal antibodies in their calves. These colostrum-derived BVDV antibodies in calves have been found to persist up to 10 months of age (24). In this particular case the antibodies could only be detected up to 3 months of age and therefore, the positive test result for BVDV antibodies in this calf at day 40 was presumed to be a result of maternal antibodies and not of an transient infection. Furthermore, no indications of a persistent infection and/or Mucosal Disease were found on serology, and histopathology failed to reveal the typical suggestive for viral encephalitis; in combination with the serology results, BVDV was deemed an unlikely causative agent in this brain anomaly. Additional tests e.g., immunohistochemistry or polymerase chain reaction on brain tissue may have provided more information to exclude this possibility with more confidence.

On post-mortem examination no obvious changes regarding the cerebellum were found microscopically but microscopic examination was performed on only a limited number of slides of cerebellar tissue. Therefore, a mild or asymmetrical cerebellar hypoplasia could have been missed and cannot be completely excluded. Furthermore, in the assessment of a possible cerebellar hypoplasia, the cerebellum to brain weight ratio could have been taken into account. Reference values for cerebellum/brain weight ratio in calves is 0.083–0.11 (25). Unfortunately, cerebellum to brain weight ratio was not determined in this case.

MRI cerebellum to total brain ratios in normal cattle and cattle affected by cerebellar hypoplasia have not been described but in dogs, the cerebellum to total brain ratio is less discriminative of cerebellar hypo-/aplasia than the brainstem to cerebellum ratio; this was reported to be 100% sensitive and specific using a cut off of 89% (26, 27). Increased conspicuity of CSF between the cerebellar folia and an enlargement of the fourth ventricle are indicative for cerebellar hypoplasia, although this is subjective and not found in all cases regarding cerebellar disease (26, 27). In this case, the MRI study identified several non-quantitative abnormalities that are in contrast to normal bovine brain anatomy as reported in Schmidt et al. (8). Future studies could focus on the value of quantitative MRI parameters in the diagnosis of CNS abnormalities in cattle.

Partial fusion of the rostral colliculi was evident in this case but there was no conclusive evidence of complete obstruction of the mesencephalic aqueduct. On post-mortem examination fusion of the rostral colliculi was confirmed, and the aqueduct was not identifiable in some sections of the midbrain and we can conclude that there was an obstructive component to the internal hydrocephalus in this case. Hydrocephalus was quantitatively confirmed using the method reported by Tsuka et al. (12). Perhaps the obstructive hydrocephalus did discomfort to the calf, however on general examination there were no indications the calf was in discomfort.

The analysis of CSF revealed an elevated TNCC characterized as a mixed pleocytosis in conjunction with a protein concentration within the reference range. CSF analysis can be useful in the diagnosis of CNS disorders however, there is little information available on the reference values of CSF analysis in bovine CNS disorders, and no specific values for calves are available (11). While age may be a factor affecting normal reference values as reported in human medicine, this has not been reported in bovids (28–30). Stokol et al. performed a study regarding CSF findings in cattle with CNS disorders (10) and TNCC was used to classify inflammation as mild (10–50 cells/μL), moderate (51–100 cells/μL) or marked (>100 cells/μL). In this particular case, based on the TNCC, “inflammation” could be postulated to have been present and can be classified as mild (10). This mild inflammation may have been linked to the congenital anomaly found in this Belgian Blue calf, specifically the encephaloclastic defect (porencephaly). Encephaloclastic defects may be associated with an intrauterine brain insult and resultant inflammatory reaction. Despite TNCC results, post-mortem examination showed no evidence for an active inflammatory process the elevated cell count in conjunction with a normal protein concentration is deemed unlikely in active inflammatory processes. It should also be noted however that CSF was collected at 3 months of age and necropsy was performed at ~10 months of age so any signs of a mild inflammatory process may have resolved. Repeated CSF analyses could have been useful in that respect but were not performed in this case, nor were serological or PCR tests performed on CSF.

With regard to other possible etiologies for the reported brain anomalies in this case, no studies for genetic mutations/defects were performed, and thus cannot be excluded. The same argument applies for possible exposure to toxins, though no evidence was found to support that differential diagnosis. Histopathology did not identify specific clues to suspect degenerative diseases or nutritional deficiencies.

## Conclusion

We reported a bovine case with congenital complex brain anomaly, detailing clinical signs and neurological examination findings, MRI characteristics and post-mortem findings. The etiology of the congenital anomaly present in this calf as described is unknown.

## Data Availability Statement

The original contributions presented in the study are included in the article/Supplementary Material, further inquiries can be directed to the corresponding author/s.

## Ethics Statement

The animal study was reviewed and approved by the Farm Animal Health clinic, Faculty of Veterinary Medicine, Utrecht University, buys farm animals with specific diseases or clinical abnormalities for educational purposes. The Farm Animal Health clinic is a closed clinic, i.e., bought animals do not return to the former owner. The calf described in this case report was also bought by the Farm Animal Health clinic for educational purposes. With every purchase, a sales contract is signed by the Faculty of Veterinary Medicine, Utrecht University, and the livestock farmer. The ethics committee (DEC, Utrecht, The Netherlands) approves the use of these animals for educational purposes (ethics approval code: AVD 108002015147). Written informed consent was obtained from the individual(s) for the publication of any potentially identifiable images or data included in this article. Identifying images of an individual are included in Supplementary Video 1. This individual (PH) has approved the use of this video by means of a written consent.

## Author Contributions

NV, KS, and PH contributed to clinical decision-making regarding diagnostics and clinical work-up. NK interpreted the magnetic resonance imaging findings. NV, KS, and NK wrote the first draft of the manuscript. All authors contributed to the article and approved the submitted version.

## Funding

All funding of this case report was provided by a general fund which is used for providing veterinary education at the Faculty of Veterinary Medicine, Utrecht University.

## Conflict of Interest

The authors declare that the research was conducted in the absence of any commercial or financial relationships that could be construed as a potential conflict of interest.

## Publisher's Note

All claims expressed in this article are solely those of the authors and do not necessarily represent those of their affiliated organizations, or those of the publisher, the editors and the reviewers. Any product that may be evaluated in this article, or claim that may be made by its manufacturer, is not guaranteed or endorsed by the publisher.
